# A Short-Armed Troodontid Dinosaur from the Upper Cretaceous of Inner Mongolia and Its Implications for Troodontid Evolution

**DOI:** 10.1371/journal.pone.0022916

**Published:** 2011-09-07

**Authors:** Xing Xu, Qingwei Tan, Corwin Sullivan, Fenglu Han, Dong Xiao

**Affiliations:** 1 Key Laboratory of Evolutionary Systematics of Vertebrates, Institute of Vertebrate Paleontology and Paleoanthropology, Chinese Academy of Sciences, Beijing, China; 2 Long Hao Institute of Geology and Paleontology, Hohhot, Inner Mongolia, China; 3 Department of Land and Resources, Linhe, Inner Mongolia, China; Raymond M. Alf Museum of Paleontology, United States of America

## Abstract

**Background:**

The Troodontidae represents one of the most bird-like theropod groups and plays an important role in our understanding of avian origins. Although troodontids have been known for over 150 years, few known derived troodontid specimens preserve significant portions of both the forelimb and the hindlimb.

**Methodology/Principal Findings:**

Here, we report a new troodontid taxon, *Linhevenator tani* gen. et sp. nov., based on a partial, semi-articulated skeleton recovered from the Upper Cretaceous Wulansuhai Formation of Wulatehouqi, Inner Mongolia, China. *L. tani* has an unusual combination of primitive and derived character states, though our phylogenetic analysis places it in a derived clade within the Troodontidae. As a derived taxon, *L. tani* has a dromaeosaurid-like pedal digit II, and this species also possesses a humerus that is proportionally much shorter and more robust than those of most other troodontids.

**Conclusion/Significance:**

The combination of features present in *Linhevenator* indicates a complex pattern of character evolution within the Troodontidae. In particular, the discovery of *Linhevenator* suggests that derived troodontids have independently evolved a highly specialized pedal digit II and have significantly shortened the forelimb over the course of their evolution.

## Introduction

Troodontids are a group of small theropods characterized by long legs and enlarged braincases [1]. Although phylogenetic analyses have varied to some extent in their placement of the Troodontidae, the majority of recent analyses place them alongside dromaeosaurids in Deinonychosauria, which in turn emerges as the sister clade to the Avialae [2]–[8]. This places troodontids in a pivotal phylogenetic position with respect to the study of avian origins. Since the discovery of the first known troodontid, *Troodon formosus,* in the Upper Cretaceous of North America in 1856 [9], troodontid specimens have been recovered from not only the Upper Cretaceous, but also the Lower Cretaceous and Upper Jurassic, of Asia and North America [1], [10]–[13]. Some fragmentary specimens have also been found in Europe [14]–[16]. So far, six troodontid species are known from the Upper Cretaceous of Mongolia and China, including *Byronosaurus jaffei* [17], *Borogovia gracilicrus* [18], *Saurornithoides mongoliensis* [19], *Zanabazar junior* [20], [21], *Tochisaurus nemegtensis* [22], and *Xixiasaurus henanensis* [23].

A few basal troodontids [12], [24]–[27] are known from relatively complete skeletons preserved in the Jurassic and Lower Cretaceous deposits of northern China, but all of the previously described derived, Late Cretaceous members of the group are represented by much less satisfactory material. The best known of these is probably *Troodon formosus*, which was originally described on the basis of a single distinctive, coarsely serrated tooth [9].The genus *Troodon* was later established as the senior synonym of *Stenonychosaurus* [28], [29]. Although *Troodon* is known from multiple specimens, many of which were originally referred to *Stenonychosaurus* [29], [30], all of the described material is rather fragmentary, and the osteology of this taxon remains incompletely known. Among other Late Cretaceous troodontids, *Saurornithoides* and *Zanabazar* are each known from a damaged skull and a fragmentary postcranial skeleton [21], as is *Xixiasaurus* [23]. *Byronosaurus* is represented by two highly incomplete mature specimens [17], [31] and two partial perinate skulls [32]. Other taxa such as *Borogovia* [18] and *Urbacodon* [33] are known from even less complete specimens.

**Figure 1:**
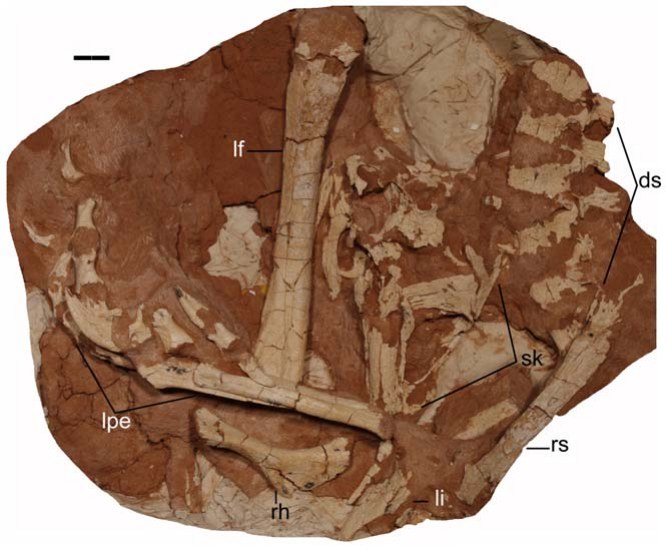
Photograph of the holotype of *Linhevenator tani* (LH V0021). Abbreviations: ds, dorsal series; lf, left femur; li, left ischium; lpe, left pes; rh, right humerus; rs, right scapula; sk, skull. Scale bar equals 20 mm.

**Figure 2:**
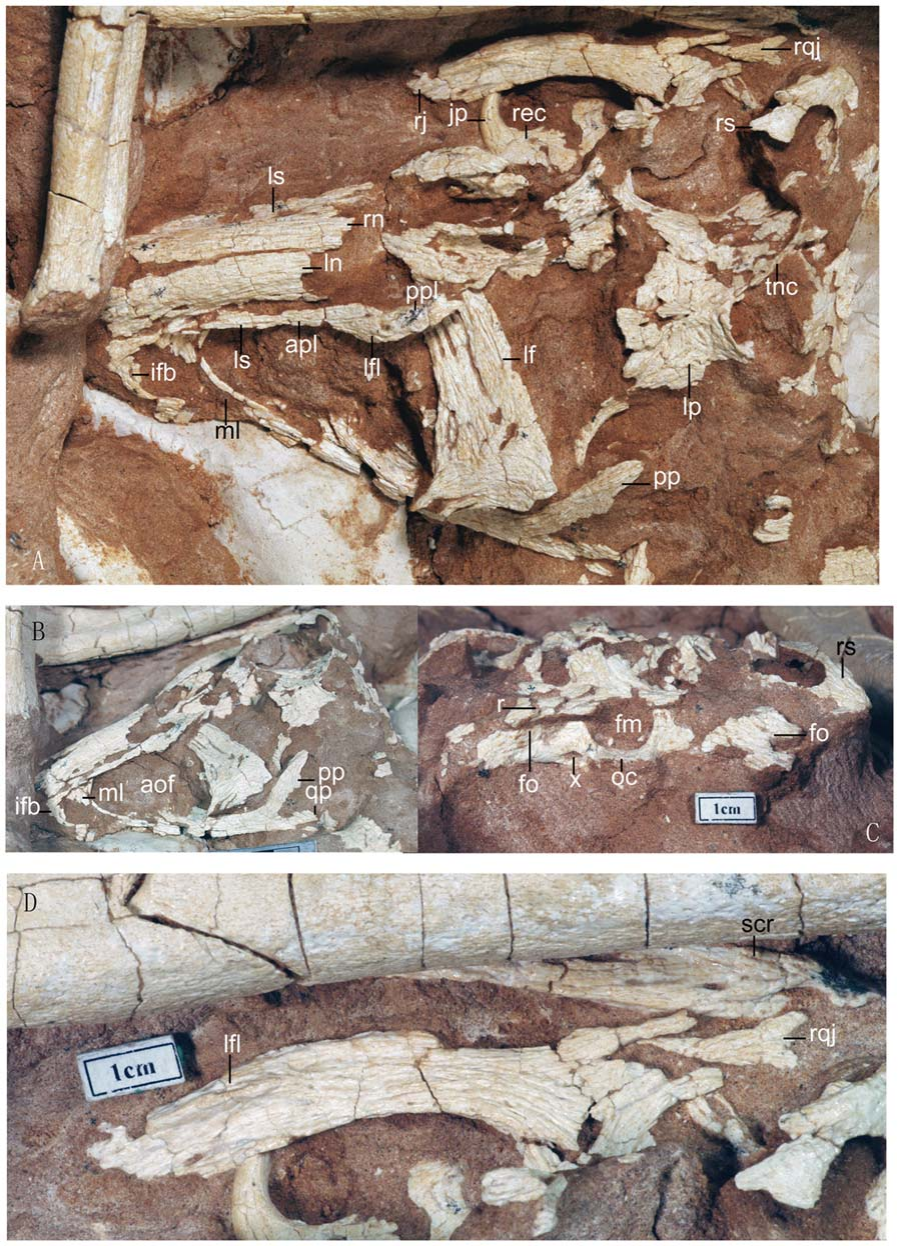
Photographs of the skull and mandible of the *Linhevenator tani* holotype (LH V0021). Skull in dorsal (A), left lateral (B), and posterior (C) views; D, right jugal, quadratojugal, and surangular in lateral view. Abbreviations: aof, antorbital fenestra; apl, anterior process of lacrimal; fm, foramen magnum; fo, fossa; ifb, interfenestral bar; jp, jugal process; lf, left frontal; lfl, lateral flange; ln, left nasal; lp, left parietal; ls, lateral shelf; ml, medial lamina; oc, occipital condyle; pp, postorbital process; ppl, posterior process of lacrimal; qp, quadratojugal process; r, ridge; rec, right ectopterygoid; rj, right jugal; rn, right nasal; rqj, right quadratojugal; rs, right squamosal; scr, surangular crest; tnc, transverse nuchal crest; X, exit for cranial nerve X.

**Figure 3:**
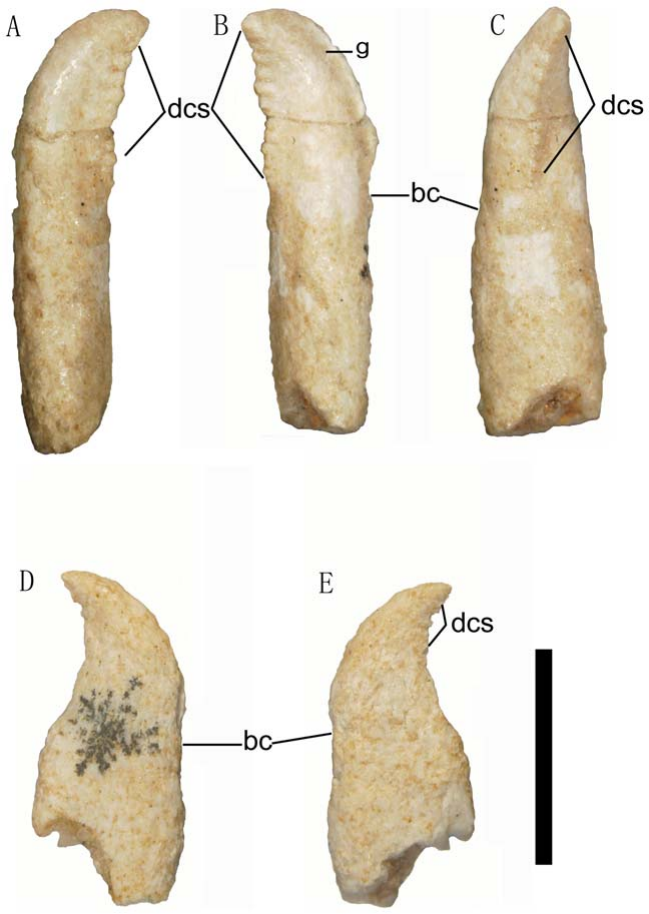
Photographs of the teeth of the *Linhevenator tani* holotype (LH V0021). Premaxillary tooth in labial (A), lingual (B), and distal (C) views; maxillary or posterior dentary tooth in labial (D) and lingual (E) views. Abbreviations: bc, basal constriction; dcs, denticles. Scale bar equals 5 mm.

**Figure 4:**
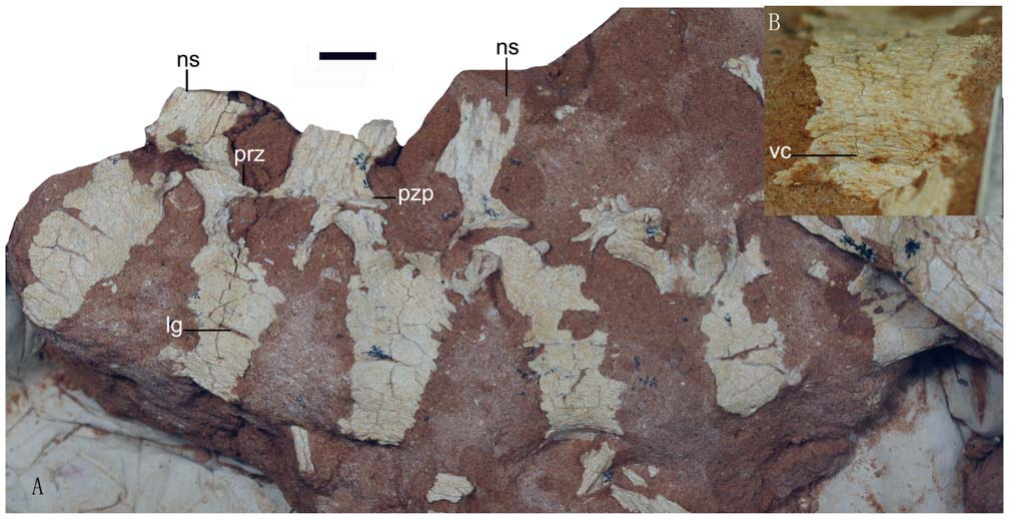
Photographs of the axial skeleton of the *Linhevenator tani* holotype (LH V0021). A, six articulated dorsal vertebrae in left lateral view; B, a middle dorsal vertebra in ventral view. Abbreviations: lg, lateral groove; ns, neural spine; prez, prezygapophysis; pzp, postzygapophysis; vc, ventral concavity. Scale bar equals 10 mm.

**Figure 5:**
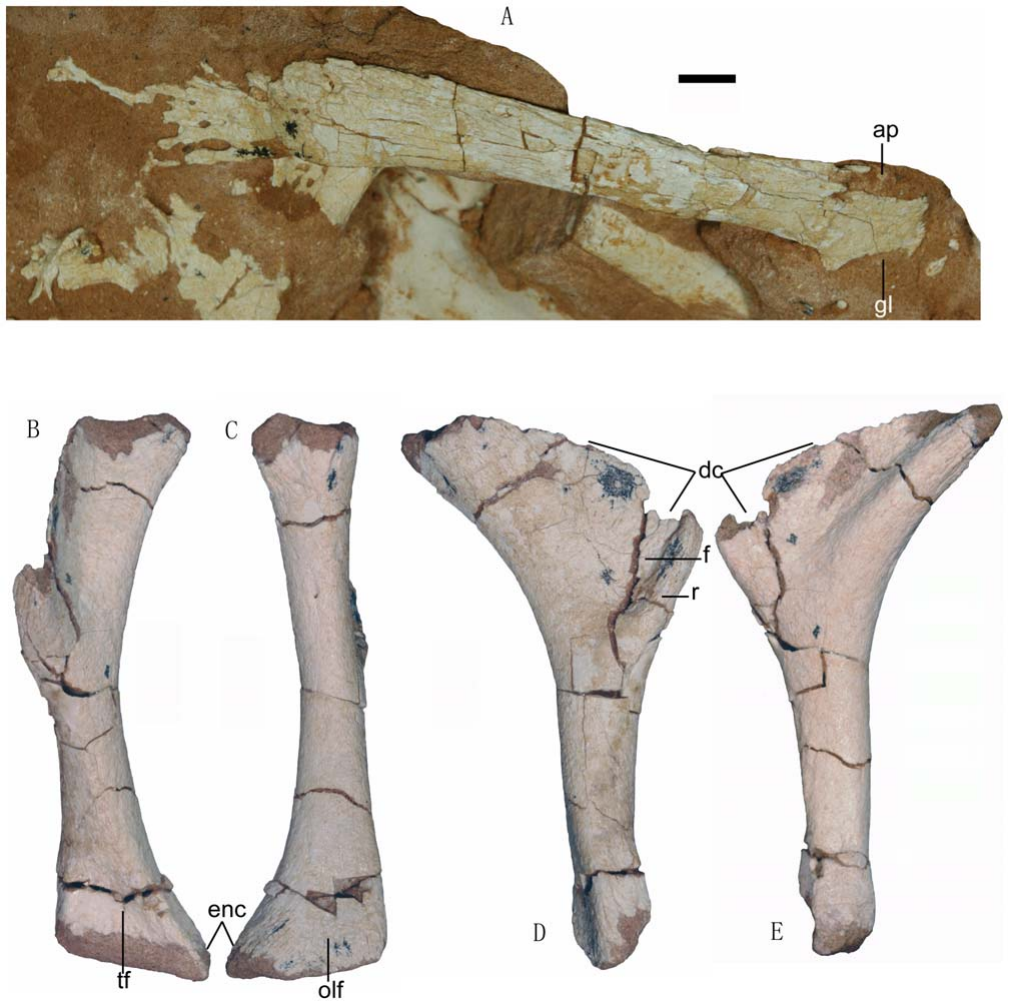
Photographs of the pectoral girdle and forelimb of the *Linhevenator tani* holotype (LH V0021). A, right scapula in lateral view; right humerus in anterior (B), posterior (C), medial (D), and lateral (E) views. Abbreviations: ap, acromial process; dc, deltopectoral crest; enc, entepicondyle; gl, glenoid fossa; f, fossa; olf, olecranon fossa; r, ridge; tf, triangular fossa. Scale bar equals 10 mm.

**Figure 6:**
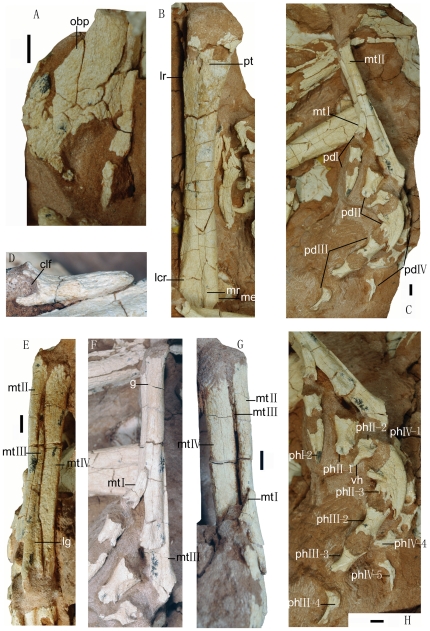
Photographs of the pelvic girdle and hindlimb of the *Linhevenator tani* holotype (LH V0021). A, left ischium in lateral view; B, left femur in posterior view; C, left pes in medial view; D, metatarsal I in lateral view; left metatarsus in dorsal (E), medial (F), and ventral (G) views; H, pedal phalanges in medial view. Abbreviations: clf, collateral ligament fossa; lcr, ridge on lateral condyle; lg, longitudinal groove; lr, lateral ridge; mr, medial ridge; me, medial expansion; mtI–IV, metatarsals I–IV; obp, obturator process; pdI–IV, pedal digits I–IV; phI-1-phIV-4, phalanges I-1 to IV-4; pt, posterior trochanter. Scale bar equals 10 mm.

Our recent series of expeditions in the Upper Cretaceous Wulansuhai Formation of Bayan Mandahu, Inner Mongolia, China, interpreted as a lateral equivalent of the Djadokhta Formation of Mongolia proper [34], has resulted in the discovery of multiple theropod taxa [35], [36]. In the present paper, we report a new derived troodontid based on a partial skeleton collected during the 2009 field season (Figures 1, 2, 3, 4, 5 and 6). Although far from complete, this specimen is nevertheless among the most intact troodontids ever to have been described from the Upper Cretaceous. It provides unprecedented data on the limb proportions of derived troodontids, and is thus important for understanding the evolutionary history of Troodontidae as a whole.

## Methods

### Fossil Collection and Preparation

A permit for field work was provided by the Department of Land and Resources, Inner Mongolia. The holotype specimen was prepared free of surface matrix, and a few bones were detached in order to better expose the cranial skeleton. Two isolated teeth, preserved in the matrix near the skull and mandible, were removed from the plaster jacket and were prepared free of matrix to permit them to be properly illustrated.

### Photography

The specimen was photographed using a digital camera (Pentax smc DAL). We also used an Olympus DP70 system to obtain digital images of the two isolated teeth through a microscope (Olympus SZX12).

### Phylogenetic Analysis

**Figure 7:**
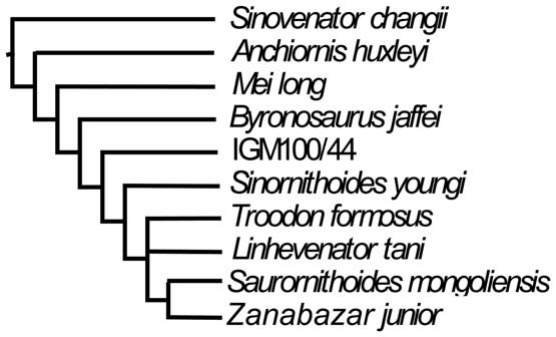
A cladogram showing the systematic position of *Linhevenator tani* among the Troodontidae, which is derived from the strict consensus of the 12 most parsimonious trees produced by our analysis of coelurosaurian dinosaur phylogeny (see Methods).

In order to assess the systematic position of the taxon described in this paper, we coded it (Table 1) into a recently published data matrix for coelurosaurian dinosaurs [37]. The matrix was analyzed using the software package TNT [38] and the analysis was run using a traditional search strategy, with default settings (starting trees: Wagner trees; swapping algorithm: TBR; 10 trees held per replicate; collapsing trees when minimum length is 0) apart from the following: 30000 maximum trees in memory and 1000 replications. All characters were unordered and none was weighted. *Allosaurus fragilis* was set as the out-group. The analysis found 12 most parsimonious trees, but the program reported that some replications had partial overflows of the tree file, leading us to carry out further branch-swapping starting from the trees in memory (“RAM”). No further most parsimonious trees were found, however. Figure 7 shows the strict consensus of the 12 most parsimonious trees (tree length  = 1303 steps, CI  = 0.34, and RI  = 0.74). A small number of features shared by the new taxon and some derived troodontids such as *Troodon* were left out of the analysis because a more comprehensive dataset on troodontid phylogeny is currently being assembled and will be published elsewhere. However, it should be noted that the signals provided by these features are consistent with our phylogenetic hypothesis, in that they support a close relationship between the new taxon and derived troodontids such as *Troodon*. As a result, our phylogenetic hypothesis would not have changed substantively if the features in question had been included in the analysis.

**Table 1:** Scorings for *L. tani.*

?0????????????????1?????1?????000???02?2??0002101?????0100??1???????????????????0?011010??0?????????01???1?0??????????????????????????0??0001????????????????????????02????11?????????????0?????????0??211011??????????00??????????0?3?01???????001001?010?1???1???????0???????1?01?????????????????????????????000????101?0110111?0????10??1?1??00001??????????0????1?????

### Nomenclatural Acts

The electronic version of this document does not represent a published work according to the International Code of Zoological Nomenclature (ICZN), and hence the nomenclatural acts contained in the electronic version are not available under that Code from the electronic edition. Therefore, a separate edition of this document was produced by a method that assures numerous identical and durable copies, and those copies were simultaneously obtainable (from the publication date noted on the first page of this article) for the purpose of providing a public and permanent scientific record, in accordance with Article 8.1 of the Code. The separate print-only edition is available on request from PLoS by sending a request to PLoS ONE, Public Library of Science, 1160 Battery Street, Suite 100, San Francisco, CA 94111, USA along with a check for $10 (to cover printing and postage) payable to “Public Library of Science”.

## Results

### Systematic Paleontology

Theropoda Marsh, 1881 [39]

Coelurosauria Huene, 1920 [40]

Maniraptora Gauthier, 1986 [41]

Troodontidae Gilmore, 1924 [10]

*Linhevenator tani* gen. et sp. nov.

#### Etymology

The generic name derives from ‘Linhe’ (area of origin) and ‘venator’ (Latin for hunter); the specific name honors Prof. Tan Lin for his contributions to the field of vertebrate paleontology in Inner Mongolia.

#### Holotype

LH (Long Hao Institute of Geology and Paleontology, Hohhot, Inner Mongolia, China) V0021, a partly articulated partial skeleton comprising skull and mandible, six anterior and middle dorsal vertebrae, right scapula and right humerus, incomplete left and right ischia, left femur, nearly complete left pes, and other fragmentary bones (Figure 1). The specimen is badly weathered; the external surfaces of the cranial elements bear numerous longitudinal grooves and ridges from surface exposure (Figure 2) and the articular ends of most of the preserved postcranial elements are eroded (Figures 3, 4, 5 and 6). The grooves and ridges probably correspond to a fibrous texture seen in some weathered bones of extant mammals [42]. Using established criteria for assessing the degree of bone weathering [42], LHV0021 can be assigned to weathering stages 2 (most limb elements) and 3 (cranial and vertebral elements) [42].

#### Locality and Horizon

Bayan Mandahu, “North Canyon” locality, Wulatehouqi, Inner Mongolia; Wulansuhai Formation, Campanian, Upper Cretaceous [34].

#### Diagnosis

Troodontid that can be distinguished from other known members of the group by the presence of the following autapomorphies: jugal with a lateral flange, surangular crest anteroventrally oriented, presence of medial expansion near distal end of femur, and wide longitudinal groove present along distal third of dorsal surface of metatarsal III.

### Description and Comparisons

The complete closure of the neurocentral sutures of all the vertebrae in which the junction between the arch and centrum is adequately preserved suggests that the holotype specimen is probably an adult individual. However, caution is warranted when relying on neurocentral fusion as a sole indicator of ontogenetic stage [43], and a more reliable assessment of ontogenetic stage should be made through histological analysis. With an estimated femoral length of about 240 mm and an estimated body mass of about 23 kg [44], the holotype specimen is a relatively large troodontid [45] (Table 2).

**Table 2:** Measurements of selected elements in *L. tani*, LH V0021 (in millimeters; * indicates estimated measurement).

Skull (snout tip to ventral end of quadrate)	220*
Three anterior dorsal centra, combined length	86*
Left scapula	170*
Right humerus	95*
Right humerus, mid-shaft mediolateral width	11
Left femur	240*
Left femur, mid-shaft mediolateral width	22
Left metatarsal I	25*
Left metatarsal II	120*
Left metatarsal III	150
Left metatarsal IV	145*
Left pedal phalanx I-1	23*
Left pedal phalanx I-2	31*
Left pedal phalanx II-1	28*
Left pedal phalanx II-2 (without the ventral heel)	14*
Left pedal phalanx II-3	50*
Left pedal phalanx III-1	40*
Left pedal phalanx III-2	29*
Left pedal phalanx III-3	25*
Left pedal phalanx III-4	31*
Left pedal phalanx IV-1	35*
Left pedal phalanx IV-2	18*
Left pedal phalanx IV-3	16*
Left pedal phalanx IV-4	17*
Left pedal phalanx IV-5	25*

Measurements are lengths except where noted.

The external surfaces of the cranial elements bear longitudinal grooves and ridges (Figures 2A and B). However, this is probably a result of weathering, especially considering that a similar texture occurs on the three preserved neural spines. The skull is estimated to be about 220 mm long, a value close to the length of the skull of the holotype of *Saurornithoides mongoliensis* [19], [21]. The snout is transversely narrow, and its dorsal surface has a marked transverse convexity, in contrast to the boxlike appearance of the rostrum in *Saurornithoides* [21]. The snout is slightly convex in lateral view as in most troodontids including the perinate *Byronosaurus* (but in contrast to the flatness of the snout in adult *Byronosaurus*) [32]. The anterolateral portion of the nasal and the anterior process of the lacrimal both contribute to a longitudinal shelf lying part way up the lateral surface of the snout (Figures 2A and B), a derived feature variably developed in other troodontids, including the basal taxa *Mei* and *Sinovenator* [25], [46]. In *Byronosaurus*, the shelf is better developed in perinate individuals than in adults [32].

The maxilla is a large element. The antorbital fenestra is much longer anteroposteriorly than high dorsoventrally (Figure 2B) and, as in other troodontids and some dromaeosaurids [36], its anterior portion is partially floored by a medial lamina. As in adult *Byronosaurus* [17], [31] and possibly *Mei*, the interfenestral bar separating the antorbital and maxillary fenestrae is flush with the lateral surface of the maxilla (Figures 2A and B). This differs from the condition seen in *Sinovenator*, *Saurornithoides mongoliensis*, *Zanabazar junior*, and perinate *Byronosaurus*, in which the interfenestral bar is inset relative to the rest of the maxilla [1], [20], [21], [32]. In *Linhevenator*, the posterior process of the maxilla has a relatively long contact with the ventral surface of the anterior process of the jugal. The posterior part of the posterior process is transversely broad (the more medial portion is embedded in the matrix), indicating the presence of a maxillary secondary palate as in other troodontids [31].

The nasal is long and transversely narrow, with parallel medial and lateral edges. The element's dorsal surface is strongly convex transversely, accounting for the arched curvature of the rostrum (Figure 2A). The anterolateral portion of the nasal is everted laterally to contribute to the lateral shelf; however, most of the lateral shelf is formed by the anterior process of the lacrimal. The anterolateral portion of the nasal bears a few small, elongate pits, which are probably of pneumatic origin.

The lacrimal has a long anterior process that terminates above the interfenestral bar. The anterior process forms the entire dorsal border of the antorbital fenestra, a feature seen in other troodontids [1], [21]. The anterior process is mediolaterally wide and dorsoventrally compressed, forming the extensive posterior portion of the lateral shelf of the snout (Figure 2A). This feature is also seen in *Sinovenator*, although in this taxon the anterior process appears to be fused with the nasal. In most other theropods, the anterior process of the lacrimal is mediolaterally compressed. The lacrimal of *Linhevenator* has a long posterior process (about 20 mm long), which is nevertheless much shorter than the anterior process (about 50 mm long). The posterior process of the lacrimal tapers posteriorly to a point, but its anterior part is expanded along the dorsolateral edge to form a lateral flange. Accordingly, the posterior process is L-shaped in cross section, with the lateral expansion of the process accounting for one arm of the “L” and the relatively deep (about 10 mm) medial surface accounting for the other arm. This feature also occurs in other troodontids [1], [46]. The medial surface of the posterior process bears a distinct groove near the dorsal edge for articulation with the frontal. The descending process is not visible in either lacrimal, but was presumably inset relative to the anterior and posterior processes as in other troodontids [46].

The jugal is a relatively slender element (Figures 2B and D). Its long anterior process has a concave dorsal margin in lateral view, as in *Zanabazar junior* [21], and a convex ventral margin. In *Saurornithoides mongoliensis*, the dorsal margin is straight [21]. In *Linhevenator* the jugal bears a longitudinal flange along the anterior half of the anterior process (Figure 2D), a feature not previously reported in any troodontid. The flange forms a flat shelf below the orbit. In *Zanabazar junior* the suborbital ramus is laterally expanded [21], somewhat similar to the condition in *L. tani*, but a shelf-like flange is not present. The ascending process is inclined posteriorly, and the dorsal half of its anterior margin forms a thick, flat surface for articulation with the descending process of the postorbital. The posterior process is relatively short, and its lateral surface is grooved to receive the anterior process of the quadratojugal. The groove terminates ventral to the ascending process of the jugal.

Small pieces of both quadratojugals are preserved. The slender, tapering anterior process of the quadratojugal fits into the groove on the lateral surface of the posterior process of the jugal (Figure 2D), and its tip is ventrally deflected. The deflected part of the anterior process looks smooth and recessed, and might have been overlapped by the quadratojugal process of the jugal. This would imply a fairly complicated interlocking contact between the quadratojugal and the jugal.

The length of the frontal is approximately double its width (Figure 2A). As in other troodontids [1], a longitudinal trough separates the orbital rim and the medial edge of the frontal. Several deep grooves are present in the trough, but these most likely represent a preservational artifact. The medial edge of the left frontal is straight and grooved. Posteriorly the orbital rim curves gently in the lateral direction to form the postorbital process, on the dorsal surface of which is a deep, transversely oriented groove for receiving the frontal process of the postorbital. This suggests that the frontal process of the postorbital is directed more dorsally than medially. The posteriormost part of the dorsal surface of the frontal is depressed to contribute to the supratemporal fossa, which, however, does not extend to the midline. Ventrally the frontal bears a large crista cranii (more than 15 mm in transverse width) to roof the orbital cavity.

The parietals are fused and form a sharp median sagittal crest between the left and right supratemporal fossae (Figure 2A). However, the crest is both low and relatively broad, particularly anteriorly, so that the two fossae are widely separated. The transverse nuchal crest extends posterolaterally, with the right and left crests forming a right angle in dorsal view. In most other troodontids [21], [46], the transverse nuchal crest is laterally oriented. The fused parietals are transversely much wider than the interorbital bar, suggesting the presence of a large braincase as in other troodontids [1].

The squamosal has a large anterior process, the lateral surface of which is deeply grooved for the squamosal process of the postorbital (Figure 2A). This groove terminates dorsal to the descending process of the squamosal. The descending process is very small and slightly inset relative to the anterior process, a feature apparently also present in other troodontids such as *Zanabazar junior*. The inset position of the descending process has been previously suggested to be an autapomorphy of Dromaeosauridae [47], in which the degree of inset is indeed much greater than in Troodontidae. The dorsal surface of the main body of the squamosal bears a depression that may be pneumatic in origin.

The axial nuchal crest (the supraoccipital crest) is barely developed, and in fact the median part of the occiput is nearly flat (Figure 2C). The paroccipital process is long, only slightly ventrally inclined, and distally blunt (Figure 2C), rather than short, considerably ventrally inclined, and distally rounded as in most other troodontids [1]. In perinate *Byronosaurus*, the paroccipital process is more laterally oriented [32]. A strong, horizontal, posteriorly concave ridge emanates from the dorsolateral corner of the foramen magnum and extends laterally to overhang a fossa on the posterior surface of the ventromedial portion of the paroccipital process. The distal end of the paroccipital process is slightly twisted in a way somewhat similar to the condition in dromaeosaurids [48]. There appears to be a slight fossa on the posterior surface of the paroccipital process near the distal end. A large foramen for cranial nerve X is visible lateral to the occipital condyle, and appears to be located in a bowl-like concavity. The foramen magnum is larger than the occipital condyle in posterior view, as in basal troodontids [1]. The foramen magnum is sub-circular in outline as in *Troodon formosus* [1], with the transverse diameter exceeding the dorsoventral one. However, these proportions probably result at least in part from dorsoventral compression of the fossil.

The ectopterygoid is a large element, with a hooked lateral process that contacts the anterior ramus of the jugal (Figure 2A). The transversely directed proximal portion of the process is long, so that the space between the hooked tip of the lateral process and the main body of the ectopterygoid is mediolaterally wide. The dorsal surface of the main body is marked anteriorly by a slight depression, which passes posteriorly into a wide groove. An even deeper fossa appears medial to the groove. These depressions are probably homologous to the dorsal recess seen in some dromaeosaurids and *Archaeopteryx* [49].

The only exposed mandibular bone is a partial right surangular (Figure 2D). The surangular has a narrow, blade-like dorsal edge, at least posteriorly. The element bears a relatively weak lateral crest which is oriented anteroventrally, rather than anteriorly as in most other theropods [46].

Two isolated teeth are preserved, one of which is identifiable as a right premaxillary tooth (Figures 3A–C). The tooth crown is robust, its labiolingual thickness being only slightly less than its mesiodistal width. The crown has a considerable posterior curvature, and the mesial and distal carinae are both displaced lingually as in the premaxillary teeth of other troodontids [1]. The mesial carina is shifted much further lingually than the distal carina. The mesial carina bears about 12 apically hooked denticles over a length of 4 mm, whereas the distal carina is unserrated. A distinct groove occurs parallel to the mesial carina on the lingual surface of the tooth crown. A similar groove has been reported in a variety of theropods, including the dromaeosaurids *Sinornithosaurus* [50], *Atrociraptor* [51], and *Deinonychus* [52], and the basal coelurosaurian *Orkoraptor* [53]. The root of the tooth is robust and thickens toward the base. The root's labiolingual thickness considerably exceeds that of the base of the tooth crown, and the root itself is also thicker labiolingually than wide mesiodistally.

The second isolated tooth is probably from either the maxilla or the posterior part of the dentary (Figures 3D and E). As in other troodontids [1], [28], the tooth crown is short apicobasally relative to its mesiodistal width, has a strong posterior curvature, and is basally constricted. These features also occur in *Archaeopteryx* [54]. The apicobasal length/mesiodistal width ratio is 1.26, similar to other troodontids but in contrast to the values of greater than 2.00 seen in most non-avian theropods. The tooth crown is much wider mesiodistally than thick labiolingually. Large denticles occur along the distal carina but are absent on the mesial carina. The mesiodistal width of the root greatly exceeds that of the crown, a feature seen in avialans [46].

Six articulated dorsal vertebrae are preserved and appear to be from the anterior to middle part of the dorsal column (Figure 4A). The centra decrease in height towards the posterior end of the articulated series: the anteriormost three centra are considerably taller than long in lateral view, the fourth centrum is somewhat intermediate, and the posteriormost two are sub-equal in length and height. The ventral surfaces of the anterior three dorsal centra are strongly compressed and keeled, whereas that of the fourth is wide and concave transversely (Figure 4B). The ventral surface is not preserved in the fifth centrum, and the sixth centrum appears intermediate in width and relatively flat on the ventral side. The lateral faces of the centra each bear a longitudinal groove about halfway up, although this feature is absent in the fourth and sixth vertebrae. The groove probably represents a reduced pleurocoel of the kind present in some basal troodontids such as *Sinovenator* [46], whereas in derived troodontids most of the dorsal centra lack pneumatic foramina [1]. In *Linhevenator* a distinct pneumatic foramen occurs immediately ventral to the transverse process of the third dorsal vertebra in the series, a feature also seen on at least the posterior dorsal vertebrae (and likely throughout the dorsal series) of *Saurornithoides*.

The prezygapophyses of the anterior dorsal vertebrae face dorsally, and the postzygapophyses face ventrally. In the posterior part of the series the prezygapophyses and postzygapophyses respectively come to face somewhat medially and laterally. A well developed diapostzygapophyseal lamina occurs on the first dorsal vertebra in the series. In the third vertebra the parapophysis is elevated to the level of the diapophysis, whereas in the other preserved vertebrae the relative positions of these structures are unclear. The neural spines are preserved only for the first three vertebrae in the series. As in some derived troodontids [1], the neural spines are considerably higher than anteroposteriorly broad.

The scapula is proportionally longer than in basal troodontids such as *Sinovenator* [46], being about 70% as long as the femur. The glenoid fossa is restricted to the ventral margin of the scapula and apparently faces ventrally (Figure 5A), in contrast to the partly lateral orientation of the fossa seen in more basal troodontids such as *Sinovenator* and *Mei* [24], [25], [46]. The slender scapular blade curves medially and ventrally, and is considerably wider distally than proximally (the distal end appears to be about twice as wide as the narrowest region of the scapular blade).

The humerus is proportionally much shorter than in basal troodontids [24], [25], [46], being only about 40% as long as the femur. It is a relatively robust element (Figures 5B–E), with the width of the distal end measuring 29% of the humeral length. Many other troodontids such as *Sinornithoides youngi* [27], [55] have a ratio smaller than 15%. The proximal half of the humerus curves strongly posteriorly (Figure 5B). A deep, subtriangular depression occurs on the posterior surface immediately distal to the proximal end of the humerus (Figure 5C), but this could be a preservational artifact. About 20 mm distal to this depression is a distinct foramen on the posterior surface of the humeral shaft, which opens partly in the proximal direction. The deltopectoral crest is large, extending for greater than 50% of the humeral length, in contrast to only about 30% in relatively basal troodontids [46], [55]. The deltopectoral crest protrudes anteriorly from the lateral edge of the shaft, and forms a prominent peak partway along its length. The part of the deltopectoral crest lying distal to the peak bears a robust, laterally projecting ridge along its anterodistal margin, a feature also present in *Troodon formosus* [1]. Proximal and posterior to this ridge is a large, sub-triangular fossa on the lateral surface of the crest (Figure 5E), a feature seen in many dromaeosaurids [36]. The distal end of the humerus is strongly expanded, particularly in the medial direction, and indeed forms a sharp entepicondylar peak. The distal end is about 2.5 times as transversely wide as the mid-shaft, compared to about 2.0 times in basal troodontids. The posterior surface of the distal end is relatively flat, but the anterior surface bears a large sub-triangular fossa.

The ischium is represented only by the middle portion of the element. It is a plate-like structure with a nearly straight posterior margin and a slightly concave anterior edge in lateral view (Figure 6A). As in relatively derived troodontids [1], the ischium has a large, subtriangular obturator process, which is located relatively distally.

The femur is relatively slender and anteriorly bowed (Figure 6B). The posterior trochanter forms a low, rounded, longitudinally elongate ridge, and is located on the posterior surface of the femoral shaft (Figure 6B) as in some derived troodontids such as *Troodon* and *Saurornithoides*, rather than on the posterolateral edge as in most other deinonychosaurs [56]. Also, the posterior trochanter extends further distally (terminating more than 70 mm distal to the proximal end of the femur) than in other troodontids, though a somewhat distally placed posterior trochanter appears to represent the normal condition in this group [57]. An extremely weak ridge near the medial edge of the posterior surface of the femoral shaft, slightly distal to the posterior trochanter, represents a reduced fourth trochanter. A relatively sharp ridge (about 20 mm long) on the lateral surface of the femoral shaft at about the same level as the posterior trochanter represents the lateral ridge. This structure is more posteriorly located than the equivalent structure in most other deinonychosaurs, though *Troodon* and *Saurornithoides* are exceptions. The distal third of the femoral shaft widens considerably in posterior view. Distally, the femur bears a wide, distally open popliteal fossa, which is bounded laterally by a thick ridge originating from the ectocondylar tuber and medially by a weaker but still distinct ridge emanating from the medial condyle. This medial ridge is located squarely on the posterior surface rather than along the posteromedial edge, partly because the femoral shaft widens slightly in the medial direction near the distal end (Figure 6B). This medial expansion is probably homologous to a prominent process on the anteromedial edge of the femur of the troodontid specimen IVPP V10597 [57].

The pes is similar in general morphology (Figure 6C) to those of other derived troodontids [1], [57]. The metatarsus is estimated to be 63% as long as the femur, proportionally shorter than in other troodontids (67% in *Troodon* and 79% in *Sinornithoides*). The arctometatarsalian metatarsus is highly compact and slender. Its length/width (measured at about mid-length) ratio is 7.3, similar to *Troodon formosus* [58]. However, many other troodontid specimens including IVPP V10597 exhibit a proportionally more slender metatarsus [18], [57], [59], [60]. The phalangeal portion of the pes of *Linhevenator* is relatively short (Figure 6C), with the combined length of the phalanges of digit III estimated to be 86% of the length of metatarsal III. A small pedal digit I is positioned medial to metatarsal II. Digit I extends approximately as far distally as does metatarsal III (assuming both digits are held straight, a convention that also applies to the following sentences). The highly modified pedal digit II extends distally almost to the level of the distal end of pedal phalanx III-2. Pedal digit IV is as robust as digit II, in terms of the mediolateral width of the phalanges, and extends beyond the distal end of pedal phalanx III-3.

Metatarsal I is small and has a tapering proximal end (Figure 6D), which lies near the mid-length of the metatarsus. Metatarsal I is dorsoventrally compressed, but its distal portion is transversely broad. The medial edge is slightly concave and the lateral edge is markedly convex. The distal end is strongly expanded dorsally, the expansion being more pronounced medially than laterally. In medial or lateral view, metatarsal I is given a slight J-shape by the abruptness of the distal expansion. On the lateral side of the distal end is a large and deep collateral ligamental fossa. As the metatarsus is preserved, the ventral rather than the lateral surface of metatarsal I is appressed to the medial surface of metatarsal II, and the lack of an articular facet on the lateral side of metatarsal I may indicate that this configuration is natural. This suggests that the phalanges of pedal digit I would have been directed laterally when dorsiflexed upon the metatarsal, rather than anteriorly as in typical non-avian theropods or posterolaterally to posteriorly as in extant birds. However, this feature is inconsistent with the condition in most other basal paravians [61], and requires confirmation from better preserved specimens.

Metatarsal II is considerably shorter than metatarsals III and IV and much more slender than metatarsal IV (Figures 6E–G), as in derived troodontids [1], [24], [46]. The proximal ends of metatarsal II and metatarsal IV contact each other, covering that of metatarsal III in dorsal view. The proximal end of metatarsal II is expanded laterally and dorsally. The shaft is dorsoventrally deeper than transversely wide, particularly over the distal half of the shaft where a weak flange protrudes ventrally. On the medial surface of metatarsal II is a distinct articular facet for metatarsal I, lying about 50 mm proximal to the distal end. The facet is slightly concave and longitudinally elongated (slightly less than 20 mm long). The distal third of the dorsal surface of metatarsal II is extremely narrow, and the distal fifth is compressed into a narrow ridge that gives the distalmost part of the shaft a sub-triangular cross-section. These features are also seen in *Troodon* [30]. The distal portion of metatarsal II is also highly compressed in IVPP V10597 [57], but not to the degree seen in the holotype of *L. tani*. As a result of this compression, the distal fifth of metatarsal II is barely visible in dorsal view in *L. tani*. The distal end of metatarsal II is minimally expanded dorsally but considerably expanded ventrally. The medial hemicondyle is more ventrally prominent than the lateral one. No distinct collateral ligamental fossa occurs on the medial side of the distal end, whereas the lateral side is concealed as a result of being appressed to metatarsal III.

Metatarsal III is the longest of the metatarsals (Figure 6E). The proximal end of metatarsal III is invisible in dorsal view, whereas the proximal and distal portions of the bone are exposed in ventral view (although the proximalmost part of metatarsal III is actually worn away). The proximal one-fourth of metatarsal III is highly laterally compressed, even strap-like, and the remainder of the bone is sub-triangular in cross section, with a ridge-like ventral surface. The distal third of the dorsal surface of metatarsal III bears a wide and shallow longitudinal groove, which extends to the distal extremity of the bone. The hemicondyles of the distal end of metatarsal III extend a considerable distance (about 20 mm) proximally on the dorsal surface. The medial side of the distal end bears a distinct but shallow longitudinally elongated fossa for ligamental attachment, whereas the lateral side is not exposed.

Metatarsal IV is the most robust of the metatarsals (Figures 6E and G), and is only slightly shorter than metatarsal III. The proximal end of metatarsal IV has a slight dorsal expansion. The proximal third of metatarsal IV is transversely wider than dorsoventrally deep, but the opposite is true for the remainder of the shaft, due to the presence of a prominent ventral flange along the lateral edge. This flange combines with the ventral expansion of the distal part of metatarsal II to form a large, longitudinal ventral concavity extending along the metatarsus, a feature seen in other troodontids and basal dromaeosaurids [62]. The dorsal surface of metatarsal IV is mostly flat, and is proximally broad but becomes transversely narrow toward the distal end of the bone.

Pedal phalanx I-1 is much wider transversely than deep dorsoventrally (Figure 6H). The distal end is considerably narrower than the proximal end in the transverse dimension. A shallow collateral ligament depression occurs on the medial side of the distal end, whereas the lateral side is poorly exposed. Pedal phalanx I-2 (ungual I) is longer than both metatarsal I and pedal phalanx I-1 and is even longer than ungual IV, whereas in basal coelurosaurs ungual I is proportionally much smaller. Ungual I is robust, only slightly curved in lateral view, and sub-triangular in cross section. The sheath groove on the medial side is weak.

Pedal phalanx II-1 is much deeper dorsoventrally than wide transversely. A proximal ventral heel, albeit a low and longitudinally extended one (Figure 6H), is present as in *Troodon formosus* [57] and *Borogovia gracilicrus* [18]. The dorsal surface of II-1 is narrow and transversely rounded, and the distal end is both dorsally and ventrally expanded. A large, relatively deep collateral ligamental fossa is visible on the exposed medial side of the distal end, in a relatively dorsal position. Pedal phalanx II-2 is much shorter than II-1 (Figure 6H), and is in fact sub-equal in length and depth as in *Borogovia gracilicrus* [18]. Proximally the phalanx bears a large, proximodistally narrow heel that protrudes strongly in the ventral direction, a feature also similar to *Borogovia gracilicrus* [18]. The medial ligamental fossa is smaller but deeper than the corresponding one on II-1 and is located on the dorsal portion of the medial surface of the distal end. Pedal phalanx II-3 (the ungual) is hypertrophied. It is much longer than II-1 and represents the longest of all the pedal phalanges, a feature seen in dromaeosaurids and some derived troodontids such as *Troodon* [30], [48], [57], [62]. However, the phalanx is nevertheless much shorter in proportion to its dorsoventral depth than the second pedal unguals of typical dromaeosaurids [63]. In contrast to ungual I, ungual II is laterally compressed and distinctly curved, and is proximally deep with a large flexor tubercle (Figure 6H). A shallow sheath groove runs along the medial side of the ungual and appears to be very wide proximally.

The second-longest pedal phalanx is III-1 (Figure 6H). A large and deep ligamental fossa occurs on the center of the medial side of the distal end. Two smaller depressions occur within the ligamental fossa, but may represent a preservational artifact. Pedal phalanges III-2 and III-3 are similar to each other in general morphology. Their proximal ends are expanded dorsoventrally, but in each case the transverse width of the phalanx at mid-shaft exceeds its dorsoventral depth. The dorsal surfaces of III-2 and III-3 are relatively flat. The collateral ligamental fossa is centrally located on the medial surface of the distal end in III-2 but more dorsally located in III-3. Ungual III is only slightly curved, and appears to be similar to ungual I in having a sub-triangular cross section.

All of the non-ungual phalanges of pedal digit IV are mediolaterally broad. This is particularly true of phalanx IV-2, which appears to be transversely wider than dorsoventrally deep. Phalanx IV-1 appears to be longer than II-1, as in dromaeosaurids and basal troodontids [63]. A deep ligamental fossa is present on the medial side of the distal end of each non-ungual phalanx of digit IV, although in the case of IV-3 only a shallow, tapering proximal extension of the fossa and a portion of the sharp proximal edge of the fossa proper are preserved. A similar proximal extension of the collateral ligamental fossa also occurs in the other non-ungual phalanges of the pes and is especially distinct in the penultimate phalanges. None of the pedal phalanges bears an extensor fossa near the distal end.

## Discussion

*L. tani* is clearly a deinonychosaur, based on the highly specialized morphology of pedal digit II. Additional evidence for the referral of the taxon to the Deinonychosauria includes the following features: antorbital fenestra partly floored by medial lamina; descending process of squamosal small, and inset relative to anterior process (in dromaeosaurids, this feature is further developed due to the presence of a large lateral shelf [45]); metatarsus with large ventral concavity bounded by longitudinal ventral flanges on metatarsals II and IV; and pedal phalanx IV-1 at least subequal in length to II-1, rather than significantly shorter. Furthermore, *L. tani* can be referred to the Troodontidae based on several troodontid synapomorphies: nasal and lacrimal form a lateral shelf on the snout [25], [46]; lateral flange overhangs descending process of lacrimal [1], [46]; anterior process of lacrimal significantly elongated to form entire dorsal border of antorbital fenestra [1], [21]; metatarsus highly asymmetrical, with short and slender metatarsal II and stout metatarsal IV.

Among troodontids, *L. tani* is more derived than basal taxa such as *Sinovenator* and *Mei* in having a fully-developed arctometatarsalian foot and a typical troodontid dentition (curved and apicobasally short tooth crowns with constricted bases and large, hooked denticles) [1], [28]. Further evidence for a derived position of *L. tani* includes the following features: dorsal neural spines considerably higher dorsoventrally than broad anteroposteriorly; proportionally long scapula; glenoid fossa restricted to ventral margin of scapula; and large subtriangular obturator process located proximal to distal end of ischium. The hypothesis that *L. tani* is a derived troodontid is consistent with the results of our numerical phylogenetic analysis, which recovered a monophyletic group composed of *L. tani*, *Troodon*, *Zanabazar*, and *Saurornithoides mongoliensis* (Figure 7). The placement of *L. tani* close to *Troodon* is further supported by a few features shared by these taxa (condition unknown in *Zanabazar* and *Saurornithoides mongoliensis*), such as a ridge along the posterolateral margin of the large deltopectoral crest and a relatively robust metatarsus. However, these features were not included in our phylogenetic analysis.

The paroccipital process of *L. tani* is long and laterally oriented, a condition reminiscent of dromaeosaurids and perinate *Byronosaurus* [32] but contrasting with the shortness and ventrolateral orientation of the paroccipital process in other troodontids, including basal ones such as *Sinovenator* [24], [46]. *L. tani* is similar to basal troodontids in having a foramen magnum that is larger than the occipital condyle, reduced pleurocoels in the dorsal centra (in derived troodontids most dorsal centra bear no pneumatic foramina [1]), and a pedal phalanx IV-1 that is at least subequal in length to II-1 (in derived troodontids pedal phalanx II-1 is considerably longer than IV-1, a condition that appears to represent an evolutionary reversal). *L. tani* also possesses a feature previously interpreted as an autapomorphy exclusive to *Byronosaurus* among troodontids, namely the fact that the interfenestral bar is flush with the lateral surface of the snout (this feature emerges in our analysis as a convergent resemblance between *Byronosaurus* and *Linhevenator*, and probably *Mei*), and likewise is similar to *Zanabazar junior* in that the suborbital ramus of the jugal is laterally expanded. Consequently, *L. tani* has a combination of primitive and derived features seen in different troodontid taxa, and its discovery further complicates the documented pattern of character evolution among the troodontids [24], [31].

Recent work on perinate *Byronosaurus* skulls has revealed significant cranial and dental changes during ontogeny in this taxon [32]. Several features seen in LH V0021 are now known to vary during the cranial development of *Byronosaurus* [32]: the rostrum is dorsally convex, as in perinate *Byronosaurus* but in contrast to the flatness of the rostrum in lateral view in adult *Byronosaurus*; the rostrum has a prominent lateral shelf as in perinate *Byronosaurus*, whereas in adult *Byronosaurus* the shelf is minimally developed; the interfenestral bar is flush with the lateral surface of the snout as in adult *Byronosaurus*, rather than inset as in perinate *Byronosaurus*; and the paroccipital process is long and slender as in perinate *Byronosaurus*, rather than short and robust as in adult *Byronosaurus*. Interestingly, LH V0021 shares more similarities with perinate *Byronosaurus* than adult *Byronosaurus*, even though LH V0021 is inferred to be an adult individual. These data have no significant effect on the systematic position of LH V0021, but provide important information on the developmental variability of some salient troodontid characters.

Particularly noteworthy is that, among troodontids, derived forms such as *L. tani* and *Troodon* (Fig. 14 in [30]) bear the closest resemblance to dromaeosaurids in the morphology of pedal digit II. In *L. tani* and *Troodon*, pedal phalanx II-1 has a slight proximoventral heel, II-2 is highly abbreviated and bears a large proximoventral heel, and the ungual is considerably larger than the other phalanges of the digit. These features are more characteristic of the Dromaeosauridae, and to a degree of other derived troodontids, than of basal troodontids, and contribute to the formation of a highly specialized second pedal digit. Probably in association with these specializations of digit II, the metatarsus of derived troodontids such as *L. tani* and *Troodon* is proportionally shorter and stouter than those of more basal troodontids. This suite of pedal features suggests that dromaeosaurids and derived troodontids may have used their pedes for predation in a similar way, resulting in the independent evolution in each case of a highly specialized second pedal digit.

Forelimb shortening occurred multiple times in theropod evolution and is best exemplified by ceratosaurs [64], compsognathids [65], tyrannosauroids [66]–[68] and alvarezsauroids [69], [70]. In each of the last two groups a continuous trend of forelimb shortening is clearly present. In ceratosaurs and compsognathids the existence of a trend is uncertain, but most members of each group have very short arms. A few other non-avian theropod groups, such as the Oviraptorosauria and Dromaeosauridae, also contain examples of short-armed taxa [71], [72], but the short-armed condition appears to characterize individual species rather than to be synapomorphic for a clade.

Previously known troodontids have proportionally shorter arms than their close relatives, the Dromaeosauridae and the Avialae, but their arms are not particularly short among non-avian theropods in general (humeral/femoral length ratio ranges from 0.52 to 0.65 in previously measured troodontid taxa). Although it is unfortunate that distal forelimb elements are not available for *L. tani*, the humeral/femoral length ratio of about 0.4 indicates that this taxon is probably among the shortest-armed non-avian theropods (for comparison, this ratio is 0.46 in the short-armed dromaeosaurid *Austroraptor* [72], 0.44 in the short-armed compsognathid *Compsognathus lingipes* [73], 0.29 in the short-armed tyrannosaurid *Tyrannosaurus rex*, and 0.35 in the short-armed abelisaurid *Aucasaurus* [74]). Because few specimens of derived troodontids preserve both the humerus and the femur, it is not known whether derived troodontids such as *Troodon* are similar to *L. tani* in having extremely short arms. Some fragmentary forearm and manual elements do appear to indicate that this is case for *Troodon* [30], which would imply that greatly reduced arms may be characteristic of a highly derived troodontid clade. This would in turn point to the possibility of a trend toward shorter forelimbs in troodontid evolution, as in the alvarezsauroids and tyrannosauroids. Such a trend would be particularly interesting given that the close relatives of the Troodontidae, the Dromaeosauridae and the Avialae, exhibit no such pattern. However, many more data on the appendicular proportions of various troodontid taxa will be needed before the hypothesis of a trend of forelimb reduction in troodontids can be adequately tested.

The diet of troodontids with coarsely denticulated teeth has been controversial, with some authors arguing that the large size of the denticles implies a degree of herbivory [75], [76] and others inferring carnivory from the detailed form of the denticles, the presence of blood grooves, and other morphological features [23], [55], [77]. If *L. tani* was a predator, the shortness of the humerus might point to a reduced role of the forelimb in capturing prey. In this connection the robust proportions of the humerus and the large size of the deltopectoral crest are intriguing, since they indicate that the forelimb nevertheless retained some functional importance. Relative to the length of the shaft, the deltopectoral crest is both longer and more prominent in *L. tani* than in basal troodontids. The crest probably was the site of insertion of the deltoideus and pectoralis musculature, as in extant birds and crocodilians [78]. The large fossa on the lateral surface of the crest is additional evidence of the insertion of the deltoid muscles. Jasinoski et al. (2006) suggested that M. pectoralis adducted and protracted the humerus in the dromaeosaurid *Saurornitholestes*, while the deltoid muscles abducted it. The two components of the deltoid complex, M. deltoideus clavicularis and M. deltoideus scapularis, would also have respectively contributed to protraction and retraction.

In *L. tani*, the considerable proportions of the deltopectoral crest and its lateral fossa are evidence that the deltoid and pectoral muscles were large. The relatively distal placement of the apex of the deltopectoral crest would have provided M. deltoideus scapularis with a large moment arm for humeral retraction, while the prominence of the crest would have given M. pectoralis a large moment arm for internal rotation of the humerus. These movements could have allowed the manus to be powerfully swung inwards or drawn posteriorly, motions with obvious potential utility in predation or in other possible activities such as digging and climbing. It is uncertain how *L. tani* actually used its forelimbs, but the size and form of the deltopectoral crest provide strong indications that the forelimbs remained functionally important despite the shortened condition of the humerus. This contrasts somewhat with the situation in the unusual dromaeosaurid *Austroraptor* [72], in which the humerus is almost as proportionally short as in *L. tani* but the deltopectoral crest is both less prominent and less distally extensive relative to the length of the humerus.
